# Neuromesodermal Progenitors: A Basis for Robust Axial Patterning in Development and Evolution

**DOI:** 10.3389/fcell.2020.607516

**Published:** 2021-01-15

**Authors:** Ramkumar Sambasivan, Benjamin Steventon

**Affiliations:** ^1^Indian Institute of Science Education and Research (IISER) Tirupati, Tirupati, India; ^2^Department of Genetics, University of Cambridge, Cambridge, United Kingdom

**Keywords:** GRN control, axis elongation, morphogenesis, posterior growth zone, tailbud

## Abstract

During early development the vertebrate embryo elongates through a combination of tissue shape change, growth and progenitor cell expansion across multiple regions of the body axis. How these events are coordinated across the length of the embryo to generate a well-proportioned body axis is unknown. Understanding the multi-tissue interplay of morphogenesis, growth and cell fate specification is essential for us to gain a complete understanding how diverse body plans have evolved in a robust manner. Within the posterior region of the embryo, a population of bipotent neuromesodermal progenitors generate both spinal cord and paraxial mesoderm derivatives during the elongation of the vertebrate body. Here we summarize recent data comparing neuromesodermal lineage and their underlying gene-regulatory networks between species and through development. We find that the common characteristic underlying this population is a competence to generate posterior neural and paraxial mesoderm cells, with a conserved Wnt/FGF and Sox2/T/Tbx6 regulatory network. We propose the hypothesis that by maintaining a population of multi-germ layer competent progenitors at the posterior aspect of the embryo, a flexible pool of progenitors is maintained whose contribution to the elongating body axis varies as a consequence of the relative growth rates occurring within anterior and posterior regions of the body axis. We discuss how this capacity for variation in the proportions and rates of NM specification might have been important allowing for alterations in the timing of embryo growth during evolution.

## Introduction

During the elongation of the embryonic body axis, multiple processes must be coordinated to ensure the generation of a well-proportioned body plan. This includes the anterior expansion of progenitor populations laid down during primary gastrulation, and the continued specification of cells from undifferentiated posterior progenitor populations. Within the posterior progenitor domain, cells transit from an undifferentiated to a differentiated state that is orchestrated by opposing signaling gradients acting at the level of the whole embryo (Diez del Corral et al., 2003; Delfino-Machín et al., 2005; Olivera-Martinez et al., 2012). In the mouse embryo, these populations undergo a stem-cell mode of growth in which a proportion of the progenitor population is retained in the posterior growth zone and continually generates derivatives in both the paraxial mesoderm and spinal cord (Nicolas et al., 1996; Mathis et al., 1999; Mathis and Nicolas, 2000). Analysis of a clonal lineage reporter that is randomly activated in a cell revealed contribution by clonal descendants to both neural and mesodermal cell types throughout the period of somitogenesis demonstrating that a subset of these progenitors are bi-potent (Tzouanacou et al., 2009). These cells reside in a region of the embryo that can continually generate axial tissues upon serial transplantation (Cambray and Wilson, 2002, 2007; Wymeersch et al., 2016), and have been termed “Neuromesodermal Progenitors” (NMPs; Henrique et al., 2015). A key characteristic of this population is their ability to maintain competence to generate both ectoderm (in this case spinal cord) and mesoderm throughout somitogenesis stages, well past primary gastrulation when much of the cells in the anterior portion of the embryo is already committed to either fate. Therefore, germ layer specification in vertebrate development continues throughout both gastrulation and posterior body elongation stages of development. The degree to which the balance of germ layer specification within the posterior progenitor domain is robust to alterations in the expansion of anterior tissues has not yet been explored but is likely important for understanding how the vertebrate body plan is established during development and altered during the evolution of vertebrates.

Broadly speaking, two alternative hypotheses exist as to what controls the balance of cell fate specification of NM competent cells, that is referred to as a balance between conditional and autonomous cell fate specification mechanisms. In one sense, cell fate specification may be occurring tissue-autonomously within a progenitor population, with the balance of outcomes dictating the proportion of cells that will either move into the posterior neural tube or into the mesoderm progenitor zone posterior to the pre-somitic mesoderm (PSM; i.e., cell fate is determined by the initial gene expression state within the progenitor population). Alternatively, cell movements within the region may determine the proportion of cells that end-up into either neural or mesodermal progenitor compartments where cells receive alternate signal exposure and only then become specified (i.e., cell fate is “conditioned” by the signals a cell receives as it is displaced into either progenitor compartment). The latter model has been supported by recent work in chick embryos, where large scale cell movements in the region have been observed to correlate with NM cell fate (Wood et al., 2019). In this review, we will outline how NM differentiation is a good experimental system in which to disentangle this complex relationship between the dynamic cell behaviors driving tissue morphogenesis and cell fate specification, and to investigate how this can generate robustness to developmental systems. We will discuss how NMPs are variable with respect to the proportions of cells within the population that undergo self-renewal both through development and between species. Despite this, we find that they maintain a distinct NM cell state that is conserved through both ontogeny and phylogeny. We conclude that this bipotent cell state generates a degree of robustness to the cell lineage variations observed as a consequence of alterations in diverse traits such as maternal-offspring trade-offs.

## A Core Gene Regulatory Network Underlies Neuromesodermal Competence

**FIGURE 1:**
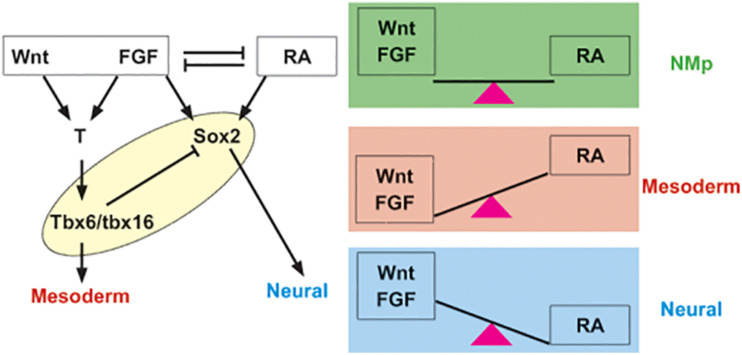
Core network regulating NM progenitor state and differentiation is invariant through ontogeny as well as phylogeny. Canonical Wnt, FGF, and RA pathway act in concert with T (Brachyury), Tbx6 and Sox2 to maintain NM state and regulate transition to neural or mesodermal fate. Note, in fish, FGF signal activates the T ortholog ntla in gastrula, but later represses it (Goto et al., 2017). Although the equation does not appear to be as simple (Garriock et al., 2015), Wnt and FGF tip the balance to mesoderm fate, while RA promotes neural differentiation (reviewed in Steventon and Martinez Arias, 2017). These cues and transcription factors considered the core network govern NM lineage throughout development and across vertebrata.

The NM regulatory network is established by an interplay of three signaling pathways, Wnt/β-catenin, FGF, and retinoic acid (RA) and the transcription factors Sox2, T (Brachyury), and Tbx6 (Figure 1). This core genetic network operates throughout axial elongation during development and appears to be a conserved regulatory unit governing NM cell fate decision making. The current model is that the bistable NM progenitor state is maintained when Wnt/β-catenin, FGF, and RA cues are finely balanced such that the neural and mesodermal outcomes are equipoised (Gouti et al., 2017; Koch et al., 2017). Fate choice is triggered by a shift in the balance among these signals. Wnt/β-catenin and FGF drive mesoderm fate, whereas RA signals tip the balance in favor of neural program (Abu-Abed et al., 2003; Li and Storey, 2011; Martin and Kimelman, 2012; Turner et al., 2014; Garriock et al., 2015; Henrique et al., 2015). Similarly, NM progenitors are characterized by the co-expression of the mesoderm T-box factor T (Brachyury) and the neural factor Sox2, suggesting that counterbalancing between the two factors may maintain the NM bipotent state (Gouti et al., 2017; Koch et al., 2017).

In addition to a role in balancing NM cell fate specification, Wnt/β-catenin signaling is also important for maintaining the progenitor state as both T and Sox2 are activated by Wnt/β-catenin signaling. In mice, Wnt3A signaling cascade directly activates T (Yamaguchi et al., 1999; Arnold and Robertson, 2009; Ramkumar and Anderson, 2011) and this regulatory relationship appears conserved between amniotes and anamniotes (Vonica and Gumbiner, 2002; Martin and Kimelman, 2008, 2009). Canonical Wnt signal also induces Sox2 in NM progenitors. In amniote embryos, Wnt-responsive Sox2 expression in NM lineage is driven by N1 enhancer harboring Lef/Tcf binding sites (Takemoto et al., 2011). In fish, sox2 is induced in the retina, a neural lineage, in response to Wnt signal (Meyers et al., 2012). While we cannot extrapolate this regulatory relationship to NMPs, Wnt/T/Sox2 appears to be a conserved regulatory unit across multiple vertebrate model organisms and we suggest that it may be fundamental to generate the NM state.

Another key element of the NM genetic network is the unit governing the exit from the bipotent progenitor state into either neural or mesodermal fates. The T-box factor Tbx6, downstream of T, regulates the transition out of NM state into paraxial mesoderm (Takemoto et al., 2011; Javali et al., 2017). Tbx6 suppresses sox2 as well as promotes paraxial mesoderm differentiation and hence, is considered a mesoderm fate switch in mouse embryos (Takemoto et al., 2011; Javali et al., 2017). In fish as well, tbx16, a paralog of mouse Tbx6, represses sox2 and therefore, favors mesodermal fate by repressing neural lineage (Bouldin et al., 2015). Whether the repression of sox2 by tbx16 is direct or requires additional regulatory interactions has yet to be fully determined. This fate choice aspect of NM network is also controlled by Wnt; Wnt/β-catenin signaling is required for the mesoderm fate choice in the NM lineage and subsequently, Wnt acts to drive mesoderm fate (Yamaguchi et al., 1999; Martin and Kimelman, 2012; Turner et al., 2014; Garriock et al., 2015). Wnt targets Tbx6 in mice (Yamaguchi et al., 1999; Dunty et al., 2008) and tbx16 in fish (Bouldin et al., 2015). The shared Wnt/Tbx6 mesoderm fate switch between amniotes and anamniotes supports a conserved core regulatory mechanism for NM specification.

Similar to Wnt, the role of FGF and RA pathways are also broadly conserved between mice and fish. In mice, FGF signal induces the expression of *T* and *Tbx6* (Ciruna et al., 1997; Naiche et al., 2011; Boulet and Capecchi, 2012). In zebrafish, while the FGF pathway induces tbx16 and suppresses the T ortholog ntla in the post-gastrula tailbud (Goto et al., 2017), it is required to activate T ortholog in the gastrula (Griffin et al., 1995). FGF signal may also regulate neural induction across vertebrates (Henrique et al., 2015). RA suppresses Wnt-driven program (Wilson et al., 2009); in mouse embryonic stem cells *in vitro*, NM state is maintained at low levels of RA, while higher levels favor neural differentiation (Gouti et al., 2017). RA arrests axial growth by negative regulation of Wnt pathway in fish as well (Martin and Kimelman, 2010). In summary, the core molecular unit regulating the NM cell specification comprising the Wnt/FGF/RA and orthologs of T/Tbx6/Sox2 appears to be an invariant feature of NM network in vertebrate embryos.

## The NM Competent State Continues Throughout Axial Elongation

While the continuity in NM regulatory network throughout axial elongation is remarkable, there are significant differences among NM cell populations across the developmental timescale. In chick and mouse embryos, the anatomy of the NMP compartment is distinct in the developing trunk and tail; the trunk NMPs are harbored in the caudal lateral epiblast and the node-streak border, while from embryonic day (E)9.5 the tail NMPare located in the chordo-neural hinge (CNH) region (Cambray and Wilson, 2002, 2007). Moreover, in mouse embryos, transcriptome analysis has revealed that the gene expression signatures are different between E8.5 trunk and E9.5 tail NMPs (Gouti et al., 2017). These changes correlate with and may underlie the differences in NMP function in the different phases of development. For example, as revealed by the clonal analysis, the extent of contribution to axial growth by the NMPs in the trunk is higher than those in the tail (Tzouanacou et al., 2009) reflecting the diminishing stem cell potential of NMPs in tail and the impending cessation of axial growth. The restriction of self-renewal potential is also observed in NMP-like cells *in vitro* upon continued culture (Edri et al., 2019) although this is likely also due to the ability to accurately recapitulate the signaling environment required to maintain the NMP population when cultured in 2D. Notably, Tbx6 expression in tail NMP compared to the rare Tbx6 + cells in the trunk NMP domain (Javali et al., 2017) may underlie the diminishing potential of tail NMPs. These key differences in anatomical territory, global gene expression and varied potential underscore that the NM progenitors in the distinct temporal compartments likely do not represent a single progenitor pool.

Although the NMP pool changes over time during ontogeny, the NM potential is a common characteristic. Loss of function mutation in the components of Wnt, FGF pathways and T-box genes in mouse embryos causes development of ectopic neural tubes at the expense of paraxial mesoderm in both trunk and tail. This argues for similar NMP to neural/mesodermal transition in trunk as well as in tail. Evidence from clonal analysis points to the presence of NM clones that undergo self-renewal and contribute differentiated cells to both the trunk and the tail axial levels (Tzouanacou et al., 2009). This idea is also supported by the ability of the progenitors to perform NM function upon heterochronic grafting (Cambray and Wilson, 2002). Moreover, anatomically, the CNH appears to be a descendant of the node-streak border (Wymeersch et al., 2016). Thus, the NM competent state is a constant feature of the progenitor compartment throughout axial elongation. Therefore, rather than describing a specific embryonic progenitor population by the term “NMP,” we propose to use it to describe a neuromesodermal competent state. We will further elaborate on this distinction before discussing its potential importance in the elaboration of species-specific body plans during vertebrate evolution.

## Cell Lineages and Cell Trajectories in Development

A particular embryonic cell population can be defined by three sets of characteristics. Firstly, its developmental potential, or the range of cell fates attainable by a cell upon directed differentiation or exposure to alternate signaling environments. Secondly, its cell lineage, or the series of mother-daughter relationships that a given progenitor cell undergoes as it progresses to its final differentiated state. Finally, the combinatorial set of genes that are expressed within a cell can be used to define a particular cell type. With the advent of single-cell sequencing technologies, this can be expanded across time to define, at a genomic level, the series of gene expression states that cells go through as they become specified to different cell states. Although this gives an impression of a lineage-like branching tree, it is a description of changing gene expression states that may or may not relate to the lineage of a progenitor population (Schier, 2020). For this reason, a distinction has been made between “kinship-lineage,” and “Waddingtonian lineage” (Marioni and Arendt, 2017). The latter refers the description of the gene expression trajectory of a cell as it becomes gradually restricted to its final cell state, in reference to CH Waddington’s visual depiction of an epigenetic landscape. For simplicity, we refer to a recent review on the interpretation of single cell RNAseq data and use the terms cell lineage when referring to kinship relationships between cells or cell trajectory when describing the series of gene expression states that cells move though during differentiation (Schier, 2020).

Using NMPs as an example, we propose that cell trajectory is an appropriate term describing the exit of cells from a bicompetent neuromesodermal state into either a neural or mesodermal progenitor state. Alternatively, cell lineage describes the mother-daughter relationships between individual NMPs that divide to make both a neural and a mesodermal progenitor cell. While the previous discussion has focussed on a shared NM cellular trajectory between anamniotes and amniotes, we will now ask whether the same is true for the conservation of a NM cell lineage.

## NMPS Have Differential Degrees of Contributions Depending on Proliferation and Volumetric Growth Rates Associated With Axial Elongation

The expansion of the NMP pool in mouse embryos is inherently linked with embryonic body axis elongation, a process that is often described as a progressive addition of cells from the tailbud in a process termed “posterior growth.” However, an analysis of proliferation rates demonstrates a uniform rate of proliferation across the PSM in both mouse and quail embryos (Bénazéraf et al., 2017; Bulusu et al., 2017). And retrospective clonal analysis using Cre-drivers specifically expressed in either myotome (Nicolas et al., 1996) or central nervous system (Mathis and Nicolas, 2000) compartments demonstrate that posterior growth is not driven solely by stem cell populations within the posterior-most region around the NMP region. Thus, while the stem-cell like clonal behaviors of dual fate neuromesodermal clones are a likely contributor to axial elongation (Tzouanacou et al., 2009), they are one of a number of cell populations in the region undergoing proliferation. Indeed, there is no strong bias of cell division in the posterior progenitor zone as compared to more anterior regions of the PSM (Bulusu et al., 2017), suggesting that there is a systemic increase in growth rates associated with posterior body elongation in the mouse. This argues against the notion of a “posterior growth zone” driving axial elongation by the continuous supply of new cells from a pool of progenitor cells in the tailbud.

**FIGURE 2:**
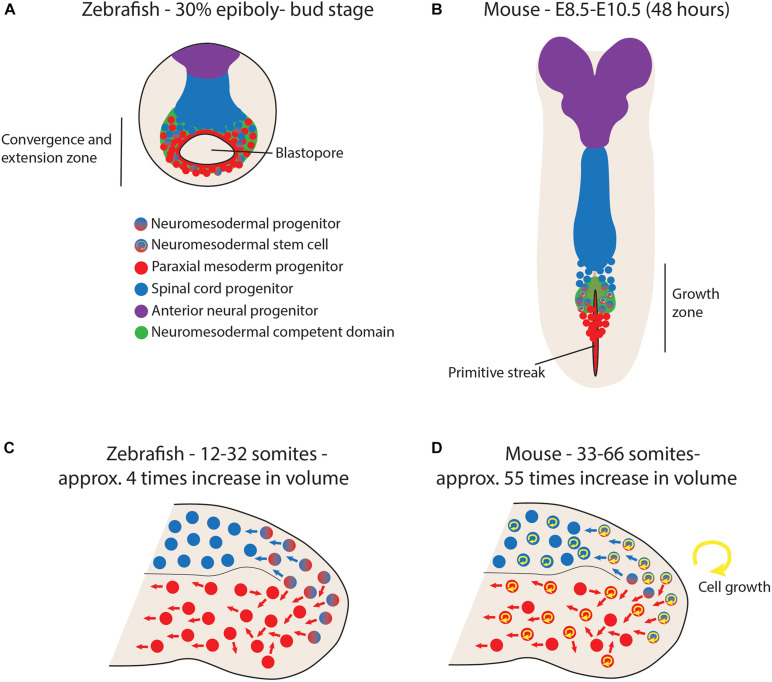
Differential growth dynamics in zebrafish vs. mouse embryos impact the stemness of neuromesodermal progenitors (NMPs). **(A)** During mid-gastrula states in zebrafish, a small proportion of progenitors within the marginal zone are bi-fated to generate either spinal cord or paraxial mesoderm. However, their rapid development with minimal growth means that few or none of these cells divide twice before gastrulation is complete, meaning that no cells can be retrospectively defined as a self-renewing stem cell. **(B)** During primitive streak closure at mid-somitogenesis stages in the mouse, a similar population of bipotent NMPs exist in the node/streak border. However, this region acts as a growth zone to continually generate new tissue as the embryo continues to grow. In this scenario, retrospective clonal analysis has revealed a stem cell mode of growth. **(C)** Upon blastopore closure in zebrafish, the tailbud is formed and contains progenitor populations capable of experimentally re-directed to either neural or mesodermal lineages upon manipulation of Wnt signaling. However, their low rates of division mean that few or none of these cells realize this bipotential during normal development. **(D)** In contrast, growth rates of over ten times relative to zebrafish result in a continuous expansion of tailbud progenitor populations. This leads to the description of mouse tailbud NMPs as a bipotent stem cell population.

Recent work in zebrafish embryos has revealed how very few dual-fate neuromesodermal progenitors exist during normal development (Attardi et al., 2018). Using a combination of CRISPR/Cas9-based genomic lineage tracing, forward fate mapping and *in toto* imaging and lineage tracing with light-sheet microscopy, two populations of neuromesodermal competent cells were lineage traced to determine their contribution to the spinal cord and paraxial mesoderm. Firstly, an early gastrula-stage population was found to have a small proportion of bi-fated progenitor cells mixed in with uni-fated progenitors. These cells are located along the anterior-most aspect of the marginal zone and have a limited period of time to enter into the prospective mesoderm compartment prior to the closure of the blastopore (Figure 2A; Attardi et al., 2018). Single cell grafts at the embryonic shield stage in zebrafish have revealed that these cells have a low probability of generating both spinal cord and mesoderm, but that Wnt signaling can push them to either fate (Martin and Kimelman, 2012). Therefore, it is likely that many cells are NM competent within this region, but that few cells realize this potential during normal development, as this would require them to divide prior to blastopore closure. As only a small percentage of cells undergo division during this time period, the number of actual NMPs during gastrulation is extremely low (Attardi et al., 2018). This is in contrast to mouse NMPs that lie within the node-streak border and caudo-lateral epiblast (Wymeersch et al., 2016). The initial NMP population is thought to arise around embryonic day 7.5 (E7.5; Wymeersch et al., 2016) and cells are able to undergo delamination and contribute to the paraxial mesoderm compartment until the closure of the primitive streak at E9.5. Therefore, early NMP populations have a much higher chance of being bi-fated in the mouse embryo, simply because mesoderm production is occurring over a longer time-period (Figure 2B). This may also mean that cells have the ability to divide multiple times before entering either neural or mesodermal compartments and would therefore be retrospectively defined as being a bipotent stem cell. This is in line with the mixed uni- and bi-fated clones observed in the trunk of the mouse (Tzouanacou et al., 2009). This difference is also mirrored in the chick embryo where long-term lineage tracing studies have revealed that a high proportion of cells with the NMP domain also contribute to both spinal cord and paraxial mesoderm (Guillot et al., 2020), and increased volumetric growth is associated with elongation of multiple axial tissues through the period of somitogenesis (Bénazéraf et al., 2017).

Upon closure of the blastopore, the tailbud forms and the cells expressing Sox2/T are situated around the dorsal-most aspect of the zebrafish tailbud (Martin and Kimelman, 2012). Mouse have a population in a similar region (Wymeersch et al., 2016) and serial transplantation experiments of the caudo-neural hinge have shown how these cells can continually give rise to both spinal cord and paraxial mesoderm (Cambray and Wilson, 2002). Elongation of the body axis from tailbud stages in zebrafish occurs largely by convergence and extension of already specified mesodermal progenitors, together with a volumetric growth of the spinal cord and notochord in an anterior-to-posterior direction (Steventon et al., 2016). In total, the entire region posterior to the 12th somite increases in volume 4-fold over this entire period. This is in contrast to the mouse tailbud that increases approximately 55-fold (Figures 2C,D; Steventon et al., 2016). Therefore, the potential for tailbud NMPs to be bipotent is greatly diminished in zebrafish due to the very low rates of proliferation within the tailbud during these stages (Bouldin et al., 2014). Indeed, no bi-fated progenitor cells exist within the tailbud during the allocation of the Sox2/T positive cells to the elongating body axis (Attardi et al., 2018). These observations are further supported by the fact that zebrafish mutants in which cell proliferation is arrested after gastrulation have no obvious disruption in posterior body elongation (Zhang et al., 2008). Therefore, there exists a considerable degree of variation in the timing of germ layer allocation when comparing across vertebrate embryos (Steventon and Martinez Arias, 2017). In this sense, the ability for a given NMP population to display stem-cell behaviors is not an autonomous characteristic of the cells themselves, but rather an outcome of the whole developing system. By altering the rates and timings of embryo growth relative to germ layer specification, a conserved neuromesodermal competent state can be elaborated in different ways depending on the system growth context in which these cells find themselves. Therefore, we must broaden our perspective beyond the molecular mechanisms driving NM fate specification so far discussed to include a consideration of the factors that influence the timing of embryo growth.

## Maternal-Embryo Trade-Offs in Evolution, and How This Impacts the Cellular Behaviors Associated With Posterior Body Elongation

The rate at which the early embryo increases in body mass must be related to the mechanism by which nutrients and uptaken and metabolized by the early embryo. Matrotrophy refers to the form of maternal nutritional provision to the embryo by the mother. In vertebrate embryos, matrotrophic input is associated with viviparity, or the retention of the developing offspring within the body of the mother until advanced stages of development. This can be contrasted with lecithotrophy in which maternal energy supplies are deposited within the egg in the form of yolk and is associated with oviparity (i.e., the release of underdeveloped offspring that can either be fertilized before or after release). Importantly, however, matrotrophy and lecithotrophy are not mutually exclusive, as many viviparous animals have a combination of yolk and direct maternal contribution (Wourms, 1972; Blackburn, 1999b). The complexity in how nutrition can be provided to the early embryo during the establishment of the body plan has a potential to generate unexpected variation in the cellular behaviors associated with posterior body elongation. For example, it might explain the variation in volumetric growth of tissues associated with these early stages of development (Guillot et al., 2020).

Viviparity has been proposed to have evolved through continued parental-offspring conflict, whereby offspring are under strong selection to increase the degree of maternal investment that they can obtain (Crespi and Semeniuk, 2004). While mothers are also under a selection pressure to increase the success of their offspring (thereby generating a general trend toward egg retention and increased investment) they do so under a conflict against their own survival and that of their additional offspring (Trivers, 1974). Among vertebrates, transitions toward viviparity has been observed at least 120 times (Crespi and Semeniuk, 2004). These include inferred transitions of around 100 in reptiles, 5 in amphibians, 9–10 in cartilaginous sharks and rays,12 in teleost fishes, and 2 in mammals (Hayssen et al., 1985; Dulvy and Reynolds, 1997; Blackburn, 1999a; Goodwin et al., 2002; Reynolds et al., 2002). Within oviparous species, further divergence exists in terms of the size of yolk supply (Cooper and Virta, 2007). The degree to which this extensive variation in maternal energy supply impacts the cellular behaviors associated in early development have not been explored. However, it raises an important question about how the mechanisms of germ layer specification, patterning and morphogenesis remain robust to these rapid evolutionary changes in the provision of the basic building blocks of cellular metabolism.

Recent studies have revealed a significant role for the regulation of metabolism in the regulation of cell fate decisions of NMPs (Bulusu et al., 2017; Oginuma et al., 2017, 2020). A gradient of aerobic glycolysis vs. oxidative phosphorylation has been observed from the posterior to the anterior regions of both chick and mouse embryos (Bulusu et al., 2017; Naganathan and Oates, 2017; Oginuma et al., 2017), with the elevated levels of glycolysis in the NMP domain reminiscent of the Warburg effect observed in cancers (Vander Heiden et al., 2020). Within the NM niche, a higher intracellular pH leads to an increase in acetylated β-catenin and a consequent activation of Wnt target genes to promote an increase in mesodermal specification (Oginuma et al., 2020). These findings open the door to further studies aimed at exploring the mechanism of interaction between developmental signaling pathways, and cellular metabolism.

Alterations in the nutritional supply to embryos as a consequence of ecological and/or evolutionary changes in development will likely impact the cellular behaviors that lead to body axis elongation in a species and tissue-specific manner. In zebrafish for example, two principle tissues contribute the most to a volumetric increase in the anterior portion of the body axis. This includes the spinal cord that progressively expands and elongates along the anterior-posterior axis (Steventon et al., 2016), and the notochord also expands through cell vacuolation (Ellis et al., 2013; Norman et al., 2018). In contrast, the PSM does not undergo a significant increase in tissue volume (Steventon et al., 2016). However, in amniotes such as the quail, the PSM also expands together with both notochord and spinal cord (Attardi et al., 2018). How alterations in nutrient supply impact these processes has not been studied, but any alterations in the rate of anterior expansion would require a counter-balance in terms of the production of additional progenitor cells from the tailbud. This raises an interesting question of how body axis proportions are maintained in such an eventuality.

We hypothesize that the NM population has an important role in allowing a degree of robustness to alterations in the expansion rates of anterior tissues. For example, if increased growth rates of neural and mesodermal populations that were specified at gastrulation resulted in an expanded pool of anterior progenitor populations, then the posterior progenitors would also have to be expanded to ensure a well-proportioned body axis. Alternatively, oviparous embryos with only a small yolk supply such as zebrafish embryos might specify a large proportion of their body axis during gastrulation, with a minimal contribution of volumetric growth. In this latter scenario, we have seen how the tailbud NMP population instead forms a reserve population of progenitors that contribute only to the final portion of the body axis (Attardi et al., 2018). By maintaining a population of uncommitted progenitor cells at the posterior aspect of the embryo, multiple aspects of their developmental dynamics can be altered to compensate for proportional changes in tissue sizes more anteriorly. This could be achieved by altering the proportional expansion of the progenitor population itself, as seen by differences in the clonal dynamics of NM populations between zebrafish, mouse and chicken embryos (Tzouanacou et al., 2009; Attardi et al., 2018; Wood et al., 2019; Guillot et al., 2020). Alternatively, it could be by altering the balance between N and M fates derived from the region and the metabolic mechanisms described above might impact this process directly (Bulusu et al., 2017; Oginuma et al., 2017, 2020). Finally, temporal shifts in the timing of NM differentiation might impact the rate at which progenitor cells are added to the embryonic axis. In the context of somitogenesis, this can have consequences to the final body plan of the embryo, as seen when comparing mouse and python embryos (Petersen and Reddien, 2009). As a consequence of Gdf11 loss of function, a prolonged expression of Oct4 has been shown to result in increase in the number of trunk vertebrae production in mouse embryos that revealed an essential network of interaction between Gdf11, Lin28 and Hox13 genes to regulate the trunk-tail transition (Aires et al., 2016, 2019). These studies raise important questions of how major transitions in body formation have been impacted during the evolution of body plans in vertebrates.

To coordinate anterior with posterior developmental processes, a mechanism is required that can transduce information across large portions of the body axis. In other words, how could it be that posterior progenitors can “sense” a differential requirement for progenitor production due to evolutionary changes in growth processes acting more anteriorly? Global alterations in the growth dynamics of multiple tissues might alter where and when NM cells and their derivatives become situated relative to the signaling sources known to be important for their differentiation. The idea that multi-tissue morphogenesis can act as an important temporal regulator of cell fate decision making has been introduced recently in the context of multi-scale timing in development (Busby and Steventon, 2020). This mechanism has been termed “tissue tectonics” to emphasize how the displacement of competent cell populations relative to sources of developmental signals and their inhibitors can impact the timing and therefore balance of cell differentiation events *in vivo*. This concept is attractive in the context of providing a mechanism for coordinating anterior vs. posterior aspects of body axis elongation as alterations in the rates of expansion in spinal cord, notochord, and/or paraxial mesoderm might directly impact the timing at which NM cells and their derivatives become spatially displaced relative to signaling centers in the tailbud. Further work into the disentangling the relative contributions of autonomous vs. conditional cell fate decision making mechanisms of NMPs will be required to test this hypothesis. In addition, appropriate comparative studies of closely related species that differ in their nutritional provision to embryos at somitogenesis stages will allow for the investigation of this hypothesis from an eco-evo-devo perspective.

## Discussion

Axial elongation mode of embryonic development, wherein the posterior structures of the animal body are gradually added from progenitors with multi germ layer potential, allows the evolution of the morphospace, especially the body length, in phylogeny. At the same time, the progenitors in the form of mesendoderm and neuromesoderm ensure proportional progenitor allocation to each germ layer and thus, ensure proportional growth of the posterior structures that require input from all germ layers. For example, contractile muscle cells and motor neurons make the fundamental functional unit required for locomotion and coupling their generation in a single developmental unit could have been advantageous. NM state provides a mechanistic link for hand-in-hand extension of the spinal cord, the skeletal muscles and the skeletal structures protecting spinal cord during axial elongation of the animal body. In addition, we have argued here that the flexibility of the dual competent NM state, in terms of the extent of contribution to either neural or mesodermal fate or with respect to the rate of growth, also confers robustness to the posterior development in the face of alterations that may be imposed upon by factors such as maternal-embryo trade off. These ideas raise the question how conserved is the NM state? The presence of cells with NM competence in basal vertebrates and at the base of chordates, i.e., in amphioxus, remains to be tested. Notably, there is growing evidence to support the deep conservation of the regulatory motif of Wnt/T across bilateria (Petersen and Reddien, 2009; Niehrs, 2010; Sethi et al., 2012; Fritzenwanker et al., 2019). How deeply the Wnt/T/Sox2 unit is conserved is unclear. Addressing these questions will lend support to the proposed significance of NM state in development and evolution.

## Author Contributions

Both authors listed have made a substantial, direct and intellectual contribution to the work, and approved it for publication.

## Conflict of Interest

The authors declare that the research was conducted in the absence of any commercial or financial relationships that could be construed as a potential conflict of interest.
